# Mutualisms weaken the latitudinal diversity gradient among oceanic islands

**DOI:** 10.1038/s41586-024-07110-y

**Published:** 2024-02-28

**Authors:** Camille S. Delavaux, Thomas W. Crowther, James D. Bever, Patrick Weigelt, Evan M. Gora

**Affiliations:** 1 Institute of Integrative Biology, ETH Zurich (Swiss Federal Institute of Technology), Zurich, Switzerland; 2 Department of Ecology and Evolutionary Biology, The University of Kansas, Lawrence, KS USA; 3 Kansas Biological Survey, The University of Kansas, Lawrence, KS USA; 4 Department of Biodiversity, Macroecology and Biogeography, University of Göttingen, Göttingen, Germany; 5 Centre of Biodiversity and Sustainable Land Use, University of Göttingen, Göttingen, Germany; 6 Campus Institute Data Science, Göttingen, Germany; 7 Smithsonian Tropical Research Institute, Panamá City, Panamá; 8 Cary Institute of Ecosystem Studies, Millbrook, NY USA

**Keywords:** Biogeography, Biodiversity

## Abstract

The latitudinal diversity gradient (LDG) dominates global patterns of diversity^1,2^, but the factors that underlie the LDG remain elusive. Here we use a unique global dataset^3^ to show that vascular plants on oceanic islands exhibit a weakened LDG and explore potential mechanisms for this effect. Our results show that traditional physical drivers of island biogeography^4^—namely area and isolation—contribute to the difference between island and mainland diversity at a given latitude (that is, the island species deficit), as smaller and more distant islands experience reduced colonization. However, plant species with mutualists are underrepresented on islands, and we find that this plant mutualism filter explains more variation in the island species deficit than abiotic factors. In particular, plant species that require animal pollinators or microbial mutualists such as arbuscular mycorrhizal fungi contribute disproportionately to the island species deficit near the Equator, with contributions decreasing with distance from the Equator. Plant mutualist filters on species richness are particularly strong at low absolute latitudes where mainland richness is highest, weakening the LDG of oceanic islands. These results provide empirical evidence that mutualisms, habitat heterogeneity and dispersal are key to the maintenance of high tropical plant diversity and mediate the biogeographic patterns of plant diversity on Earth.

## Abstract (web summary)

The effect of the latitudinal diversity gradient on plants on oceanic islands is weakened by classical abiotic physical drivers of island biogeography and, to a greater extent, by mutualism with other species.

## Main

The latitudinal diversity gradient (LDG) is among the most pervasive biogeographical patterns in ecology, with species richness generally increasing with proximity to the Equator^1,2^. The LDG is particularly strong for terrestrial plants, which serve as the focus for most hypotheses regarding the mechanisms driving high levels of tropical diversity^1,2,5–8^. The relatively large area of tropical regions, high heterogeneity, long-term climate stability and the accumulation of host-specific pathogens and competitors are long-standing hypotheses to explain the high diversity of these plant communities^9–13^. In addition, recent work highlights the potential for positive interactions to contribute to the LDG, but the role of these mutualistic interactions remains untested^10,14^. Here we leverage an exception to the LDG for plants on oceanic islands to disentangle some of the ecological mechanisms driving the LDG at the global scale.

**Extended Data Fig. 1:**
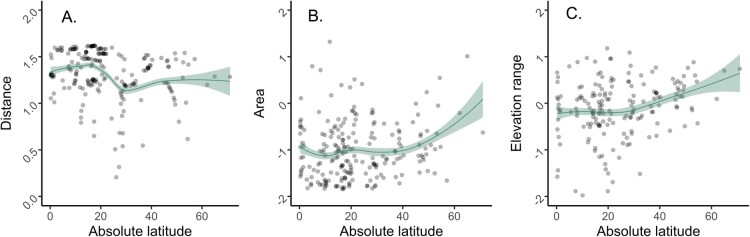
Relationships between absolute latitude and distance, area, and elevation range. Island isolation (distance, A), area (B), and elevation range (C) plotted against absolute latitude, with a smoothed localized regression line shown in teal. Points represent raw data, with shaded area representing 95% confidence intervals. Source Data

The physical abiotic characteristics of islands influence plant colonization, speciation and extinction rates^4^. Isolation from mainland regions generally limits immigration, whereas reduced land area and narrower elevational ranges on islands are associated with lower habitat heterogeneity and higher extinction rates^4,15,16^. Collectively, reduced area, reduced elevation ranges and increased isolation can contribute to limiting species richness on islands relative to mainlands (hereafter referred to as the island species deficit). If these abiotic filters have similar proportional effects on island species richness across latitudes (for example, if 100 km of distance causes a 10% reduction in island species richness across latitudes), then the absolute species deficit will be greatest near the Equator due to the high diversity of mainland species. However, islands also tend to be more isolated^17^ and smaller near the Equator^18^, which could amplify distance and area effects at low absolute latitudes, and potentially dampen the LDG on islands even further (Extended Data Fig. 1). If these classic island biogeographical filters are strong enough, then their effects could cause islands to diverge from the global LDG trend.

In addition to these physical limitations, recent work has shown that island floras are further constrained by the dispersal limitation of mutualists^19–23^, including pollinators^21,23^, arbuscular mycorrhizal (AM) fungi^19,20^ and nitrogen (N)-fixing bacteria^22,24^. Plants associating with these mutualists are underrepresented on islands relative to mainlands due to a mutualism filter, further contributing to the island species deficit. Importantly, high plant diversity in tropical mainland sites is composed almost entirely of plant species that rely on biotic pollinators^23^, AM fungi^19,25–27^ and/or N-fixing bacteria^28^ (hereafter biotically pollinated plants, AM plants and N-fixing plants, respectively), which suggests that mutualisms contribute disproportionately to the island species deficit in tropical ecosystems and that prior studies have underestimated the importance of the mutualist filter at lower latitudes. The collective effects of these mutualists on the LDG remain unexplored. Given their variation in size, isolation and biotic colonization, oceanic islands represent a unique model system to disentangle the biotic and abiotic factors that drive the latitudinal gradient in plant diversity at the global scale.

Here we explore the strength of the LDG among vascular plant communities on oceanic island ecosystems worldwide relative to mainlands. We calculate the relative contribution of different biotic and abiotic filters driving the island species deficit across the globe. We do this using plant inventories from the Global Inventory of Floras and Traits (GIFT) v3.0 database^3^ for 345 islands (212 oceanic islands) and 608 mainland regions ranging from 57° S to 81° N. Specifically, we hypothesize that (1) plant assemblages exhibit a weaker LDG on oceanic islands compared to mainlands, (2) both abiotic drivers and the mutualism filter mediate the resulting island species deficit, and (3) each group of mutualistic plant species, including biotically pollinated, AM and N-fixing plants, contributes disproportionately to the island species deficit at low latitudes, thereby weakening the LDG of oceanic islands. Our results provide compelling evidence that there is a reduced LDG on oceanic islands and that this is caused, in part, by the disproportionate filtering out of mutualistic plant species at lower latitudes.

## The LDG is weakened on oceanic islands

**Fig. 1:**
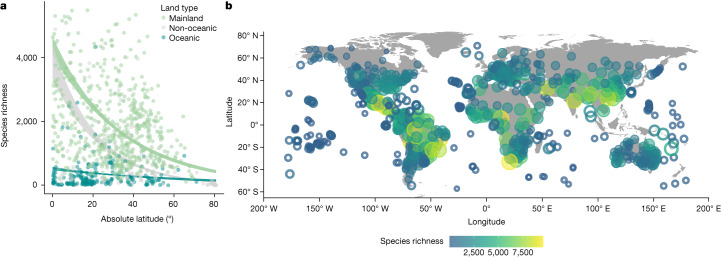
Oceanic islands show a weakened LDG. The global LDG is strong for mainland floras, but weakened on oceanic islands (*n* = 939 biologically independent floras, *z*-value = 3.47, *P* < 0.001; Supplementary Table 1). **a**, Mainlands are shown in green, oceanic islands are shown in turquoise and non-oceanic islands are shown in grey. Lines represent model predictions, with shaded area representing standard errors. Dots show raw data. **b**, Mainland data are shown superimposed on grey continents, with centroids as filled circles. Oceanic island data are shown as open circles. Source Data

**Fig. 2:**
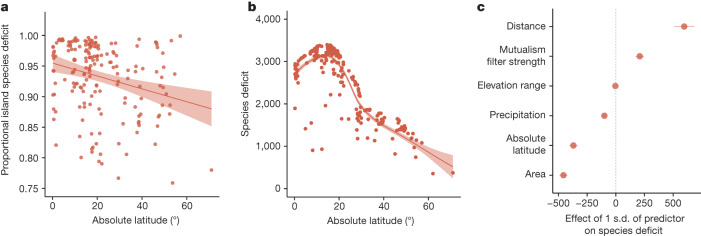
Island species deficit is influenced both by abiotic drivers and a mutualism filter. **a**, The proportional island species deficit (1 − ratio of island to predicted mainland species richness for each island) declines with increasing absolute latitude (*t*(207) = 3.381, *P* = 0.00086), confirming that the cumulative effect of all filters on island plant species richness increases with proximity to the Equator. **b**, The reduction of the LDG on oceanic islands is the result of a greater species deficit (that is, mainland species richness minus island species richness at equal latitudes) at low absolute latitudes compared with higher latitudes (*n* = 212 biologically independent floras). Lines in **a** and **b** represent linear regression (lm function in R) and local regression (locally estimated scatterplot smoothing) fits, respectively, the shaded area represents standard errors and dots show raw data. **c**, This species deficit is influenced by classic island biogeographical variables, such as area and distance, and the strength of the mutualism filter (*n* = 212 biologically independent floras; Supplementary Table 2). Statistical analysis by Satterthwaite’s approximations for *t*-test and corresponding *P* values. The measure of centre (*x* axis) represents the effect of 1 s.d. of each predictor (*y* axis) on the species deficit and error bars represent standard errors, with greater distance from the 0 line indicating a stronger negative or positive effect (see Extended Data Fig. 3 for variance explained). Source Data

**Extended Data Fig. 2:**
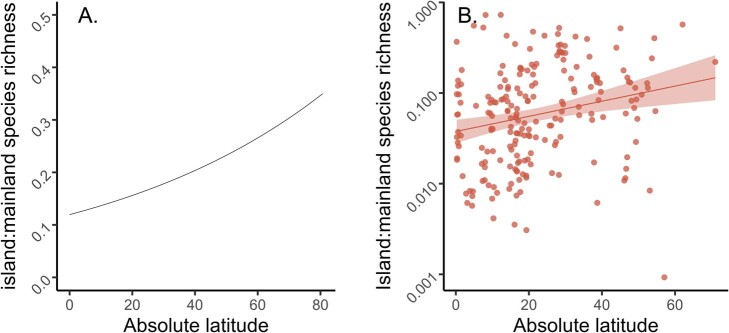
Evidence for stronger deficit at low latitudes with null model. The ratio of predicted island to mainland species richness (A, predicted values extracted from GLM models used to test for the LDG on mainlands and islands, Supplementary Information Table 1A) and for each island (B) increases with increasing latitude, confirming that the cumulative effect of all filters on island plant species richness increases with proximity to the equator. Shaded areas in B represent 95% confidence intervals. Source Data

Our results show that oceanic islands exhibit a significantly weaker LDG (Fig. 1 and Supplementary Table 1, interaction *z*-value = 3.47, *P* < 0.001; oceanic island model *z*-value = −2.97, *P* = 0.003) relative to mainlands (*z*-value = 18.66, *P* < 0.001). The weaker LDG of islands supports observations from previous studies^29^. However, by separating out the trends on oceanic and non-oceanic islands, we reveal the strength of this global dampening effect, as oceanic islands display a fundamentally different LDG. Specifically, we observed an almost twofold reduction in the slope of the LDG from mainlands to oceanic islands (mainland slope = −0.029; oceanic island slope = −0.015), whereas this trend was not apparent for non-oceanic islands, which showed no dampening of the LDG. In fact, the LDG of non-oceanic islands is about 2.8 times stronger than that of oceanic islands (non-oceanic island slope = −0.044; non-oceanic × mainland interaction *z*-value = −4.30, *P* < 0.001). Using two distinct null model approaches, we confirm that these patterns cannot be explained by a constant reduction in species richness among islands relative to mainland plant communities (Fig. 2a, Extended Data Fig. 2 and Supplementary Table 1E). The marked dampening of the LDG on oceanic islands relative to mainlands highlights that the island species deficit is stronger in tropical regions and can provide fundamental insights into the mechanisms that drive the global LDG in plants.

## Drivers of the island species deficit

The weakened LDG on oceanic islands results from a high island species deficit in tropical regions (Fig. 2b). We calculated the species deficit for each island by subtracting observed richness on that island from predicted mainland species richness at the same latitude (extracted from the mainland generalized additive model, deviance explained: 18.7%). We then explored how variation in the island species deficit was explained by absolute latitude, island isolation (distance from mainland), area, elevation range, precipitation and mutualism filter strength estimated as the proportion of mainland flora composed of plant species associating with at least one of the three mutualist types we assessed (biotic pollinators, AM fungi, or N-fixing bacteria) at the same latitude using Akaike information criterion (AIC) model averaging. We subsequently tested whether the effects of each significant predictor varied with latitude and mutualism filter strength by adding pairwise interactions that improved model fit in sequence.

**Extended Data Fig. 3:**
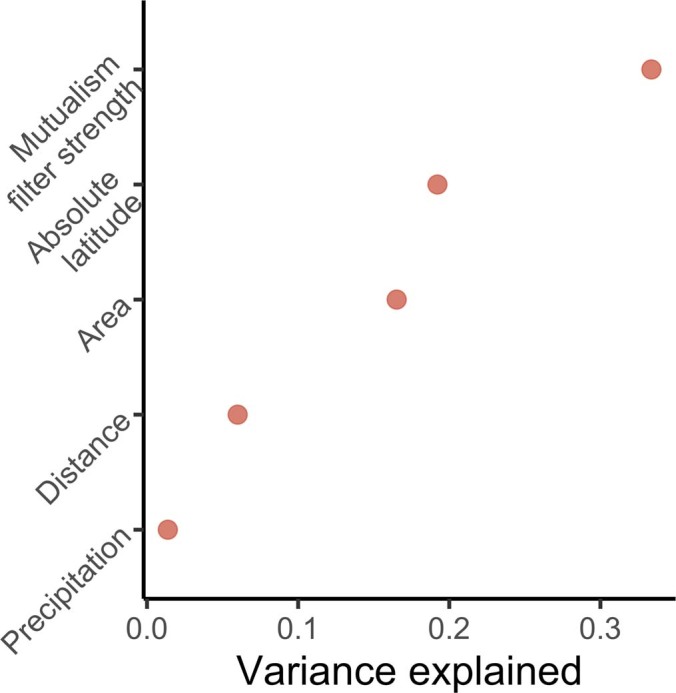
Island species deficit is influenced by both abiotic drivers and a mutualism filter. The island species deficit is influenced by classic island biogeographical variables, such as area and distance, and the strength of the mutualism filter. The mutualism filter explains the largest amount of variation in the island species deficit, followed by latitude and area. Source Data

Abiotic drivers contributed substantially to the island species deficit (effect sizes in Fig. 2c, Extended Data Fig. 3 and Supplementary Table 2). Variance partitioning indicated that collectively, these abiotic variables explained 24.5% of the total variation in the island species deficit (area: 14.3%, distance: 4.8%, precipitation: 1.2%, elevational range: 4.2%). As expected from island biogeography theory^4^, smaller and more isolated islands exhibited a greater species deficit (area: *z* = 12.98, *P* < 0.001; distance: *z* = 6.78, *P* < 0.001). Reduced dispersal to more distant islands and the lower habitat heterogeneity of smaller islands likely contributed to their greater species deficits. Unexpectedly, the elevational range of islands—also a proxy for habitat heterogeneity—was not associated with the species deficit (*z* = 5.06, *P* = 0.57). Climate was also important; the species deficit decreased with increasing total precipitation (*z* = 5.07, *P* < 0.001), suggesting that wetter conditions were more favourable to plant colonization.

Mutualism filter strength was the strongest individual predictor of variation in island species deficit (25.0% of total variation explained). Moreover, when accounting for additional variation explained by inclusion of mutualisms (interactions between mutualism filter strength and abiotic drivers), the total variation explained by mutualism filter strength increased to 28.5%. The species deficit increased where more mainland plant taxa engaged in mutualisms with pollinators, AM fungi and N-fixing bacteria (*z*-value = 5.55, *P* < 0.001). When testing the interactions between mutualism filter strength and classic abiotic island biogeographical variables, we found compounding effects of the mutualism filter strength with declining area (*t*(203) = −3.4, *P* < 0.001) and increasing island distance (*t*(203) = 2.54, *P* = 0.012). Together, these results suggest that mutualism filters are a dominant factor shaping the weakened LDG on oceanic islands and, correspondingly, a key factor contributing to high diversity in the mainland tropics.

## Contributions of each mutualism type

**Fig. 3:**
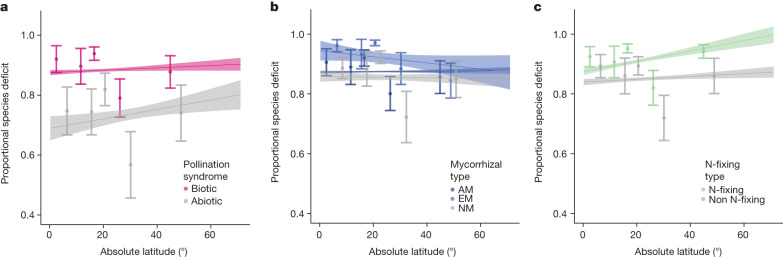
Evidence for a mutualism filter for each mutualism examined. **a**–**c**, Each mutualism type (pollination syndrome, mycorrhizal and N-fixing) shows a biotic mutualism filter on island establishment, with proportional species deficit higher for biotically pollinated (**a**; *P* = 0.0001), AM (**b**; *P* = 0.0382) and N-fixing (**c**; *P* < 0.0001) plant species across latitude (data binned to facilitate visualization, *n* = 212 biologically independent floras). Note that proportional species deficit increased slightly towards the Equator (absolute latitude × N-fixing status interaction: *t*(415) = 2.06, *P* = 0.04). Statistical analysis by Satterthwaite’s approximations for *t*-test and corresponding *P* values. Lines represent model predictions and shaded areas represent standard errors (Supplementary Tables 3–5). Each set of points and error bars refer to the mean and 95% confidence interval for each of 5 sections of 20% of the raw data. Source Data

**Extended Data Fig. 4:**
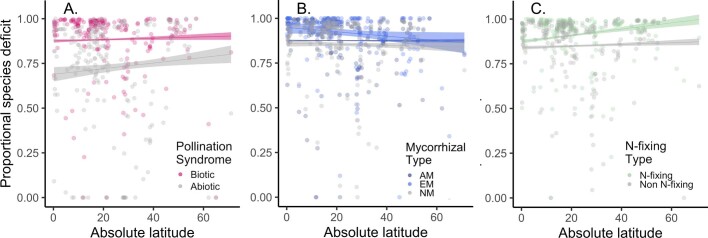
Evidence for a mutualism filter for each mutualism examined. Each mutualistic type (pollination syndrome, A; mycorrhizal, B; and N-fixing, C) shows a biotic mutualism filter on island establishment, with proportional species deficit higher for biotically pollinated, AM, and N-fixing plant species across latitude. Lines represent model predictions, with shaded area representing standard errors. Points represent the raw data. Source Data

By separating the effects of biotic pollination, mycorrhizal fungal and N-fixing bacterial associations, our analyses provided evidence that all three mutualism types contribute to the weakened island LDG. Biotically pollinated plants (*t*(415) = 7.37, *P* < 0.001), AM plants (*t*(835) = 2.07, *P* = 0.03), ectomcorrhizal (EM) plants (*t*(835) = 3.25, *P* = 0.0012), and N-fixing plants (*t*(415) = 7.56, *P* < 0.001) were underrepresented on islands relative to equal latitude mainland communities (proportional species deficit) compared to abiotically pollinated, NM, or non N-fixing plants, respectively (Fig. 3 and Extended Data Fig. 4). Specifically, biotically pollinated plants, AM plants and N-fixing plants showed 16%, 2.1% and 6.3% higher species deficits, respectively, when compared with non-mutualist plants. These results affirm the generality of previous analyses showing filters due to biotic pollination^23^, AM fungi^19^ and N-fixing bacteria^22^, and suggest that the mutualist filter is due to the reduced likelihood of simultaneous co-colonization of partners rather than particularities of dispersal mechanisms of mutualists. Moreover, these results confirm the operation of the mutualism filter for island species deficits based on modelled mainland species richness for each individual island. The effect of N-fixing plant species on proportional island species deficit increased slightly towards the Equator (absolute latitude × N-fixing status interaction: *t*(415) = 2.06, *P* = 0.04). We lack the data needed to robustly assess the three-way mutualist status of each species missing from islands, but expect the effect of the mutualism filter to be even greater for plants with multiple mutualisms, as previously shown for plants that associate with both AM fungi and N-fixing bacteria^22^.

**Fig. 4:**
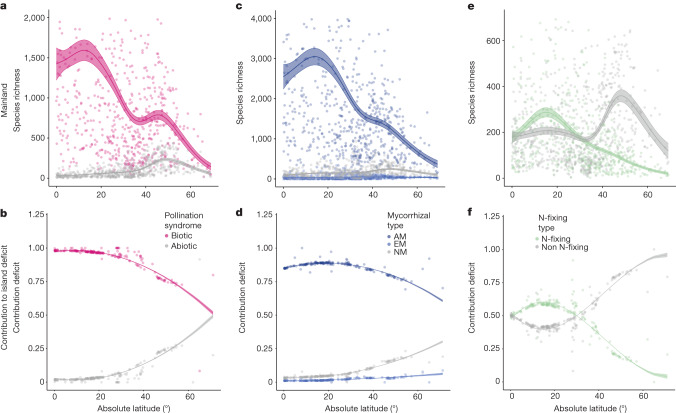
Mutualistic plant species contribute disproportionately to the island species deficit at low latitudes. **a**,**c**,**e**, Species richness of each mutualistic type shifts latitudinally across mainlands, with low absolute latitudes dominated by biotically pollinated (**a**), AM (**c**) and, to a lesser degree, N-fixing (**e**) plant species (Supplementary Tables 3–5). **b**,**d**,**f**, The contribution of each mutualist status within each mutualist type to species deficit shifts with latitude as the overwhelming contribution of mutualistic plant species at low latitudes decreases at higher absolute latitudes. This greater contribution to species deficit by biotically pollinated (**b**), AM (**d**) and N-fixing (**f**) plant species at low absolute latitudes, in conjunction with evidence of a mutualism filter for each group (Fig. 3), indicates a mechanism explaining the reduction of the LDG among oceanic islands. Lines represent model predictions, shaded areas represent standard errors and points are raw data. Source Data

The total effects of each mutualism filter on island diversity are multiplied by the relative frequency of each mutualism type across latitude (Fig. 4). Because of their high tropical diversity, biotically pollinated and AM plant species contributed more than 95% and nearly 85%, respectively, of the total species deficit among lower latitude islands (Supplementary Tables 3 and 4, *P* < 0.001). Our model predicted that the island with the smallest absolute latitude in our dataset (0.24°) lost 1,419, 2,156 and 85 species owing to the biotic pollination, AM and N-fixing mutualist filters, respectively. These contributions declined at higher latitudes, with biotically pollinated plants and AM plants contributing less than 60% of the species deficit at a latitude of 70° (Fig. 4b,d). N-fixing plant species showed the same general trend of a decreasing contribution to species deficit with increasing latitude (*P* < 0.001), but their contributions to the island deficit only exceeded non-N-fixing plants up to an absolute latitude of 30° (Fig. 4e,f), likely because of their distinct mainland abundance patterns. Finally, we confirmed that interactions with abiotic factors did not meaningfully alter the patterns of disproportionate contribution to species deficit by mutualistic plants (Supplementary Tables 3–5). Collectively, these patterns suggest that mutualistic plant species—particularly those that associate with biotic pollinators and AM fungi—contribute disproportionately to the species deficit at low latitudes, consistent with the hypothesis that mutualism filters contribute substantially to the weakened LDG on islands.

## Consequences of a weakened island LDG

Our results show that oceanic islands experience a weakened LDG and highlight the importance of both abiotic and biotic drivers in generating this trend. Specifically, our analyses affirm the importance of classical LDG hypotheses, including island isolation and size, in predicting species richness. However, we also demonstrate the importance of mutualisms in shaping plant diversity. Until now, research relating the plant LDG to biotic interactions has mostly focused on negative interactions, especially those with herbivores and pathogens^9,10,30,31^. Although the mutualism filter has not classically been considered to be a component of LDG theory, these findings support previous work highlighting that many plant species are unlikely to colonize islands in the absence of the mutualists on which they rely^10,14,24^. In fact, we find that mutualistic filters can be as important as classic physical features in reducing the LDG on oceanic islands. As such, our findings contribute to a growing body of work recognizing the important role of plant-associated mutualists, including mycorrhizal fungi^32,33^ and pollinators^34^ in plant biogeography, highlighting their likely role in the maintenance of diversity and shaping the LDG across the globe.

Our analyses focus on plants, but these patterns are likely to have broader implications for the maintenance of diversity of other taxa. The limitation of plant species associated with mutualists implies that mutualists themselves may be limited by their plant hosts, potentially generating a weakened LDG in these affected groups. If this is the case, the three mutualist types examined here may also show a weakened LDG on islands. Moreover, the mutualists, consumers and pathogens of plants, which were not addressed in our analyses (that is, seed dispersers, specialist herbivores and pathogens), are also likely to experience repercussions of this mutualism filter. The cumulative effects of mutualistic filters are likely to cause plant communities and networks of interactions to be less complex on islands than their mainland counterparts, which may explain higher invasion in these systems^35,36^ (but see ref. ^37^). Overall, we expect that the patterns presented here only scratch the surface of mutualism contributions to diversity and community complexity and urge expanded research on this topic.

**Extended Data Fig. 5:**
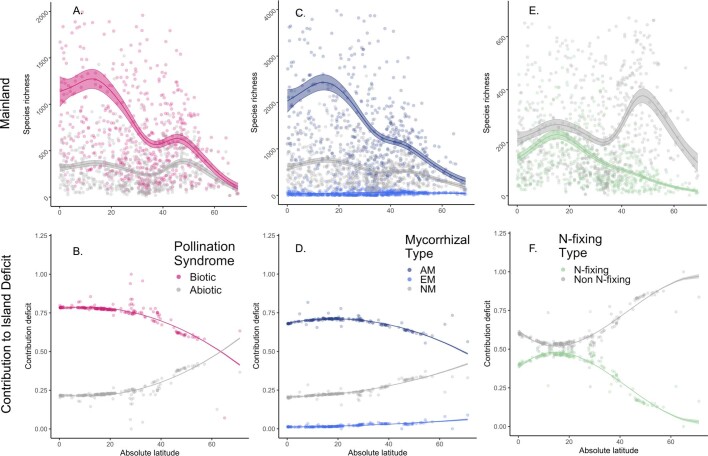
Sensitivity analysis confirms that mutualistic plant species contribute disproportionately to the island species deficit at low latitudes. Species richness of each mutualistic type shifts latitudinally across mainlands, with low latitudes dominated by biotically pollinated, AM and, to a lesser degree, N-fixing plant species (A, C, E, Tables S3–S5). The contribution of each mutualist status within each mutualist type to species deficit shifts with latitude as the overwhelming contribution of mutualistic plant species at low-latitudes decreases at higher latitudes (B,D, F). This greater contribution to species deficit by biotically pollinated, AM, and N-fixing plant species at low latitudes, in conjunction with evidence of a mutualism filter for each group (Fig. 2), indicates a mechanism explaining the reduction of the latitudinal diversity gradient among island plant communities. Lines represent model predictions, with shaded area representing standard errors; points are raw data. Source Data

In dissecting the weaker LDG on oceanic islands, our work highlights the fundamental role of mutualisms in structuring the LDG among plant assemblages. However, there are multiple areas for future improvement of these studies. Here, mutualistic status was derived from the literature and may be inconsistent across all occurrences of a species or genus (that is, evolution of mutualistic status may have occurred on either mainlands or islands), with most data being collected from mainland regions^38^. Therefore, expanding mutualist status databases would substantially benefit the field. However, our main findings are robust to a sensitivity analysis that assigns 20% of mutualist-associated plant species in each assemblage to non-mutualist-associated plant species (Extended Data Fig. 5). Further, our deficit calculation relies on a model of latitudinal diversity among mainlands, and is therefore imprecise and does not account for regional variability in mainland diversity or area. Future work could benefit from explicitly pairing islands with their sources to improve measures of species deficit^23^. Finally, we did not evaluate how all hypothesized drivers of the LDG contribute to the island species deficit or if the factors identified here act in concert with other variables to maintain diversity.

In conclusion, we show that abiotic island biogeographical factors and plant mutualisms interact to weaken the LDG on islands. The richness of plant species on the average oceanic island in equatorial regions is relatively similar to that of oceanic islands in high latitudes, representing a marked divergence from the global pattern on mainlands, where tropical species richness can be hundreds of times higher than in boreal regions. Along with the smaller size and isolation of islands, this work emphasizes the critical importance of species interactions in the production and maintenance of plant diversity. In particular, these patterns demonstrate that mutualisms have a key role in shaping global trends in diversity, and could be as important as the well-studied contributions of plant pathogens and herbivores to high tropical diversity^39–41^. Ultimately, these distinctions of plant assembly patterns on islands provide unique insights into the fundamental ecological mechanisms that drive variation in plant diversity worldwide.

## Methods

### Datasets

Regional plant distribution data along with explanatory variables were extracted from the GIFT v3.0 database^3^ using the GIFT R package^42^. The GIFT database integrates plant distributions from floras and regional checklists with geographic, environmental and socioeconomic data; it includes data for nearly 3,400 regions, over 350,000 plant species, and about 4 million species-by-region occurrences. This database is unique in that it includes many island regions in addition to mainland regions. Here we used all GIFT regions for which checklists of native angiosperms were available. We removed islands for which island geology (that is, volcanic, floor, shelf, fragment and others) was undetermined, and condensed island types into two major types: oceanic (including atoll, floor or volcanic) or non-oceanic (shelf or fragment). Oceanic islands represent newly formed land masses, assumed to be colonized de novo, whereas non-oceanic island have some historical connection to mainlands, and therefore direct contact with these source populations. Explanatory variables included absolute latitude and longitude of the region’s centroid, area (in km²; log-transformed for all analyses), and elevational range^43^ (difference between lowest and highest elevation in m above sea level). When elevation range was unknown or reported as zero from aerial elevation maps, we assigned an elevation of 1 m as a minimum necessary elevation for data collection (that is, the island must be above sea level to be detected). For islands, we also included island isolation, or distance to the nearest mainland^44^ (km). Prior to analyses, we removed islands smaller than 6 km^2^, to remove any small island effect^45–47^ (that is, small islands exhibiting a weaker species–area relationship than larger islands).

We considered three mutualisms—pollination syndrome, mycorrhizal status and N-fixing status—as they are known to influence plant establishment and fitness^48–50^ and have the most complete data. Determination of mutualist status for pollination, mycorrhizal, and N-fixing mutualisms followed similar approaches. Pollination syndrome status for each plant species was extracted from the GIFT database as either biotic or abiotic pollination. Pollination syndrome was assigned to a given species first by matching species-level data^51,52^ (using https://ecotraits.landcareresearch.co.nz/, http://tropical.theferns.info and https://www.floraweb.de); if not possible, we relied on family level assignment^53,54^. Mycorrhizal status was assigned using the FungalRoot database^38^. We assigned mycorrhizal status based on species-level status when possible. However, if species-level assignment was not possible, we applied genus-level assignment to the given species. We included four major mycorrhizal types: AM, EM, orchid mycorrhizal (ORC) and NM plant species. We assigned ambiguous (AMNM) or dual mycorrhizal types (AMEM)—those plant species that have been found as exhibiting either (AMNM or AMEM) or both mycorrhizal statuses (AMEM)—to AM as they are capable of forming these mycorrhizal types and may therefore experience the AM filter^19^. Our assignment of pollination status and mycorrhizal status to unknown species based on family and genus level, respectively, is valid as these mutualism types have been shown to be phylogenetically conserved at these taxonomic scales^23,55^. Finally, N-fixing status was assigned using the most recent N-fixing plant database from Werner et al.^56^. We first assigned N-fixing status (either N-fixing or non-N-fixing) at the species level; if this was not possible, we assigned a family level proportion to the remaining species in a given assemblage^22^. In total, we assigned 256,499 out of 880,176, 388,505 out of 1,816,146, and 7,645 out of 318,926 species to species-level assignment for pollination, mycorrhizal and N-fixing status, respectively. Note that the total number of species vary depending on whether information for genus or family was available for each mutualism status; we removed unmatched species for which family- or genus-level mutualist status was unknown.

In order to address how differences in resolution of assignments might affect our main conclusions, we conducted a sensitivity analysis. Specifically, for each mutualism type, we relabelled 20% of the mutualist-associated plant species as non-mutualists, with 20% of each flora relabelled from biotically pollinated to abiotically pollinated, N-fixing to non-N-fixing, or arbuscular mycorrhizal to non-mycorrhizal. We then reran models testing for island species deficit and contribution to species deficit (see ‘Statistical analyses’ for more details). Our major conclusions are robust to this sensitivity analysis, with the contribution to species deficit remaining highest for mutualism-associated plant species at low latitudes, and the relative importance of the mutualism filter emerging as a driver that is at least as important as abiotic drivers (Extended Data Fig. 5).

### Statistical analyses

We tested for the plant LDG and evaluated whether this pattern was weaker on oceanic islands than mainlands (hypothesis (1)). We modelled species richness within each mutualism type (pollination syndrome, mycorrhizal and N-fixing) for each mutualist status (biotically or abiotically pollinated; AM, EM, ORC or NM; N-fixing or non-N-fixing) on mainlands based on absolute latitude. We then used these models to predict expected species richness for each mutualist status for each island based on mainland communities at the same latitude. We used these mainland-based predictions and observed island floras to calculate total species deficit, proportional species deficit, and contribution to species deficit for each mutualist status on each island. We then tested for drivers of the difference between island and mainland species richness at equal latitude (species deficit, extracted from mainland model), including both abiotic variables and an aggregate metric of proportion of mutualistic plant species on the mainland (mutualism filter strength, hypothesis (2)). Finally, we tested for the role of mutualist status within each mutualism type, latitude, and other biogeographical variables in predicting both proportional species deficit and contribution to species deficit (hypothesis (3)). All models included a residual autocovariate to correct for spatial autocorrelation^57^, with neighbourhood distance set to 2,000 km and weighted by inverse distance. All analyses were done in R 3.4.1^58^, with linear mixed models constructed with the lme4 package^59^; significance for these models was tested using lmerTest^60^, using Satterthwaite’s approximations for *t*-test and corresponding *P* values, with a *P* value of <0.05 used as the threshold for significance.

To model the LDG across our dataset, we used a generalized linear model (GLM) with negative binomial errors and a log-link function. The response variable was species richness (number of species); the fixed effect predictors were land type (mainland, oceanic island or non-oceanic island), absolute latitude, and an interaction between absolute latitude and land type. This analysis tested for a significant weakening of the LDG on each island type relative to the mainland (interaction between absolute latitude and land type in initial model). Results did not change qualitatively if we also included area in these models. However, we directly assessed the effect of island area in our subsequent deficit models; therefore, we did not include it here. We then reran models within each land type to examine the strength of the LDG in each land type separately.

We implemented two null approaches to confirm that the dampened oceanic island LDG cannot arise from a filter applied equally across all latitudes. We first generated an oceanic island null model to directly test using the model described above. To do this, we took individual mainland values of richness and multiplied them by the ratio of island to mainland species richness at the lowest latitude island (ratio = 0.12 at 0.02° latitude). This approach forces both intercepts of the oceanic island and null oceanic island to be equal; therefore, deviation from the null model can only be detected at higher latitudes. We found that oceanic islands exhibited a significantly weaker LDG than this null oceanic island model (Supplementary Table 1). Second, we examined the ratio of island to mainland species richness predicted from the LDG model across latitude. We find that the ratio increases with increasing latitude, more than doubling over 60° of latitude (Fig. 2a and Extended Data Fig. 2), confirming that the cumulative effect of all filters on island plant species richness increases with proximity to the Equator.

Our three metrics of island (1) species deficit, (2) proportional species deficit and (3) contribution to species deficit were determined by comparing observed island richness to the predicted species richness of an equal latitude mainland community. We used generalized additive models^61^ to model the relationship between latitude and species richness for total mainland species richness and for each mutualistic status within each mutualism type (biotically or abiotically pollinated; AM, EM, ORC, or NM; N-fixing or non-N-fixing). We predicted mainland species richness using a smoothed term of absolute latitude (with no limits on *k*) with a negative binomial error distribution and a log-link function. We used these models to estimate the expected species richness of a mainland community at the same absolute latitude of each island for each group. We then used observed and expected values of total species richness for each island community to calculate the island species deficit (expected species richness based on mainland generalized additive model minus observed total species richness), proportional species deficit (the proportion of expected species richness that was not observed on each island, within each of the eight mutualist statuses; for example, AM proportional deficit = (AM expected – AM observed)/AM expected), and the contribution to total species deficit (species deficit of each mutualist status divided by total species deficit of a given island; for example, AM contribution to species deficit = (AM expected – AM observed)/(total species richness expected – total species richness observed)). Species deficit (1) represents the raw difference between island and equal latitude mainland species richness, proportional species deficit (2) the proportion of mainland species lost from an equal latitude island within a particular mutualist status, and the contribution to species deficit (3) represents the relative contribution of a particular mutualist status to the total species deficit.

To model species deficit, we used a weighted GLM with Gaussian errors and an identity link (i.e. no transformation); sampling units were weighted by their sample size (expected species richness based on latitude). We included the fixed effects of absolute latitude, area, distance, elevation range, precipitation, and mutualism filter strength. The mutualism filter strength variable represented the proportion of mainland plant species that are mutualistic with at least one of the three mutualist types we assessed (biotic pollinators, AM fungi, or N-fixing bacteria). To assess relative importance of different variables, we subsequently used (1) AIC model averaging to determine the significance of each variable included in our model and (2) variance partitioning for the final model from model selection using the relaimpo package^62,63^. Finally, we ran additional models to test interaction terms that we hypothesized may be important a priori. Specifically, we tested interactions between mutualism filter strength and each abiotic driver (area, distance, precipitation, and elevational range) as well as absolute latitude, followed by interactions between absolute latitude and each abiotic driver. We accomplished this by sequentially adding interactions, and we only retained terms that improved model fit, as determined by AIC values.

Our approach to estimating proportional and contribution to species deficit produced implausible estimates for a few islands with unusually high species richness (occurring in pollinator, mycorrhizal, and N-fixing analyses on 17, 41, and 2 out of 212 islands, respectively). Rather than removing these observations, we constrained these extremes to the smallest and largest values that should occur for proportional species deficit and contribution to species deficit (0 and 1; ‘constrained response’ models). We report results from this approach because it provided the best fit to the data. However, we also confirmed that this approach did not influence our results by rerunning the analyses including these extreme values (‘extreme response’ models) and using a linear model with Gaussian errors and the same predictors as we report in this manuscript. All results are reported in Supplementary Tables 3–5.

To model proportional species deficit, we used a weighted GLM with Gaussian errors and an identity link. A separate model was run for each mutualism type (pollinator syndrome, mycorrhizal and N-fixing). Fixed effects included absolute latitude, area, distance, elevation range, precipitation, mutualist status and the interaction between absolute latitude and mutualist status. Region was specified as a random effect to control for repeated measures of each island (that is, the separate measures of each level of mutualist status). These models allowed us to test for a consistent biotic mutualist filter within each mutualism type and evaluate whether this varied with latitude. To do this, we used post hoc contrasts using pairwise comparisons of model coefficients with least squares means.

To model contribution to species deficit, we used a weighted GLM with Gaussian errors and an identity link. We included the fixed effects of absolute latitude (as a polynomial function), mutualist status (either pollinator syndrome, mycorrhizal type or N-fixing type), area, distance, elevation range, and the interactions between mutualist status and absolute latitude. Region was again specified as a random effect. To confirm that these patterns were not influenced by interactions between latitude and other biogeographical variables, we separately modelled each mutualist status within each mutualist type with a more complex set of predictors. We created full models that added interactions between absolute latitude and area, distance, precipitation, and elevational range, and we used stepwise model reduction to simplify these models based on significance (*P* < 0.05) to obtain the most parsimonious model. For both proportional species deficit and contribution to species deficit, we confirmed that alternative modelling approaches (such as beta regression or binomial errors with a logit link) did not produce a better fit to the data.

## Online content

Any methods, additional references, Nature Portfolio reporting summaries, source data, extended data, supplementary information, acknowledgements, peer review information; details of author contributions and competing interests; and statements of data and code availability are available at 10.1038/s41586-024-07110-y.

### Supplementary information

Supplementary Information

### Source data

Source Data Fig. 1

Source Data Fig. 2

Source Data Fig. 3

Source Data Fig. 4

Source Data Extended Data Fig. 1

Source Data Extended Data Fig. 2

Source Data Extended Data Fig. 3

Source Data Extended Data Fig. 4

Source Data Extended Data Fig. 5

## Data Availability

Source data are provided with this paper.

## Code availability

All code and data used to complete analyses for the manuscript are available at https://github.com/c383d893/IslandLDG. Data analysis was conducted and visualizations generated in R (v. 4.3.2) using the following packages: mgcv (v1.8.41), gridExtra (v2.3), betareg (v3.1.4), MASS (v7.3.58.1), lme4 (v1.1.31), lmerTest (v3.1.3), lsmeans (v2.30.0), ggeffects (v1.1.4), spdep (v1.2.7), ggplot2 (v3.4.0), ncf (v1.3.2), ape (v5.6.2), sjPlot (v2.8.12), gridExtra (v2.3), MuMIn (v1.47.1), maps (v3.4.1), sf (v1.0.9), car (v3.1.1), viridis (v0.6.2), tidyverse (v1.3.2) and GIFT (v1.3.0).
