# Supplementary material for: Mutualisms weaken the latitudinal diversity gradient among oceanic islands
Source: Nature. 2024 Feb 28;627(8003):335–9. doi: 10.1038/s41586-024-07110-y (PMC10937366; doi:10.1038/s41586-024-07110-y)
Supplement: Supplementary file 1 — Supplementary Information [file 41586_2024_7110_MOESM1_ESM.pdf]

nature portfolio

https://doi.org/10.1038/s41586-024-07110-y

Supplementary information

Mutualisms weaken the latitudinal diversity gradient among oceanic islands

In the format provided by the authors and unedited

Mutualism filters, habitat heterogeneity, and isolation weaken the latitudinal diversity gradient among oceanic island floras

Camille S. Delavaux[1,2], Thomas W. Crowther[1], James D. Bever[2,3], Patrick Weigelt[4,5,6], Evan M. Gora[7,8]

[1]*Institute of Integrative Biology, ETH Zurich (Swiss Federal Institute of Technology), Universitätsstrasse 16, 8092 Zurich, Switzerland.*

[2]*Department of Ecology and Evolutionary Biology, The University of Kansas, 2041 Haworth Hall, 1200 Sunnyside Avenue, Lawrence, Kansas 66045, USA*

[3]*Kansas Biological Survey, The University of Kansas, 106 Higuchi Hall, 2101 Constant Ave, Lawrence, Kansas 66047, USA*

[4]*Department of Biodiversity, Macroecology & Biogeography, University of Gottingen, Büsgenweg 1, 37077 Göttingen, Germany*

[5]*Centre of Biodiversity and Sustainable Land Use, University of Göttingen, Göttingen, Germany*

[6]*Campus Institute Data Science, Göttingen, Germany*

[7]*Smithsonian Tropical Research Institute, Panamá City, Panamá.*

[8]*Cary Institute of Ecosystem Studies, Millbrook, New York, USA*

Table of Contents

SI Table 1..4
SI Table 2..4
SI Table 3..4-5
SI Table 4..5
SI Table 5..5-6

SI Table 1 | Model results for the latitudinal diversity gradient in plant species on mainlands and islands.

Model results for GLMs used to test for the LDG on mainlands and islands. The significance of the interaction between land type, in particular between mainlands and oceanic islands, and absolute latitude in (A) confirms that although there is a relationship between absolute latitude and species richness on mainlands, this is weakened on oceanic islands. Separate tests for the LDG on mainlands (B) and both island types separately (C, D) confirm that absolute latitude predicts a stronger reduction in species richness for mainlands and non-oceanic islands relative to oceanic islands.

SI Table 2 | Model results for drivers of island species deficit.

Model results for models used to test for drivers of island species deficit, including the influence of abiotic variables and the mutualism filter strength, including model averaging (A) and subsequent model selection via testing increasing interactions (B). The variable 'mututalism filter strength' here represents the proportion of mainland floras associating with any one of the three mutualisms included in our analyses (biotic pollination, arbuscular mycorrhizal, or N-fixing).

SI Table 3 | Model results for proportional specific deficit and contribution to species deficit for pollination mutualisms.

Model results for GLMs testing drivers of proportional species deficit and contribution to species deficit within the pollination mutualism as well as contribution to species deficit within

mutualist status to include interactions with distance, area, and precipitation (removed in final model if non-significant). Models are run for dataset in which extremes were constrained to 0 or 1 ("constrained response model", reported in main models, A.), or where extremes were kept ("extreme response model", B.).

SI Table 4 | Model results for proportional specific deficit and contribution to species deficit for mycorrhizal mutualisms.

Model results for GLMs testing drivers of proportional species deficit and contribution to species deficit within the mycorrhizal mutualism as well as contribution to species deficit within mutualist status to include interactions with distance, area, and precipitation (removed in final model if non-significant). Models are run for dataset in which extremes were constrained to 0 or 1 ("constrained response model", reported in main models, A.), or where extremes were kept ("extreme response model", B.).

SI Table 5 | Model results for proportional specific deficit and contribution to species deficit for N-fixing mutualism.

Model results for GLMs testing drivers of proportional species deficit and contribution to species deficit within the N-fixing mutualism as well as contribution to species deficit within mutualist status to include interactions with distance, area, and precipitation (removed in final model if non-significant). Models are run for dataset in which extremes were constrained to 0 or 1 ("constrained response model", reported in main models, A.), or where extremes were kept ("extreme response model", B.).

Source Data 1 | Source data for all figures of the manuscript.

Source data is provided for all figures (1-4) and extended data figures (1-5).

Mutualism filters, habitat heterogeneity, and isolation weaken the latitudinal diversity gradient among oceanic island floras

Supplementary Information

SI Table 1 | Model results for the latitudinal diversity gradient in plant species on mainlands and islands

Model results for GLMs used to test for the LDG on mainlands and islands using. Statistical analysis used Satterthwaite's approximations for t-test and corresponding p values. The significance of the interaction between land type, in particular between mainlands and oceanic islands, and absolute latitude in (A) confirms that although there is a relationship between absolute latitude and species richness on mainlands, this is weakened on oceanic islands. Separate tests for the LDG on mainlands (B) and both island types separately (C, D) confirm that absolute latitude predicts a stronger reduction in species richness for mainlands and non-oceanic islands relative to oceanic islands. Finally, a null model analysis (D) assuming a constant proportion of the mainland species are capable of colonizing islands confirms that the island slope is significantly different from this null expectation.

A. Mainlands v. Islands (df = 946)				
Variable	Estimate	Std. Error	z value	p value
(Intercept)	8.8287	0.0654	135.0570	<2.00E-16
landtype (non-oceanic)	-0.1204	0.1468	-0.8200	0.41206
landtype (oceanic)	-2.1238	0.1111	-19.1210	<2.00E-16
absolute latitude	-0.0287	0.0019	-15.3380	<2.00E-16
spatial autocorrelation	1.2069	0.0438	27.5350	<2.00E-16
landtype (non-oceanic)*absolute latitude	-0.0154	0.0036	-4.2950	0.00002
landtype (oceanic)*absolute latitude	0.0133	0.0038	3.4650	0.00053
B. Mainlands (df = 605)				

(Intercept)	8.6818	0.0547	158.8000	<2.00E-16
absolute latitude	-0.0269	0.0015	-17.6200	<2.00E-16
spatial autocorrelation	0.7883	0.0422	18.6600	<2.00E-16
C. Oceanic Islands (df = 209)				
(Intercept)	6.4529	0.1167	55.2940	<2.00E-16
absolute latitude	-0.0115	0.0039	-2.9670	0.00300
spatial autocorrelation	1.5455	0.0906	17.0590	<2.00E-16
D. Non-oceanic Islands (df = 130)				
(Intercept)	9.1585	0.2031	45.0940	<2.00E-16
absolute latitude	-0.0569	0.0043	-13.2410	<2.00E-16
spatial autocorrelation	0.6513	0.1321	4.9290	0.00000
E. Null model (df = 1552)				
(Intercept)	8.2920	0.0399	208.0220	<2.00E-16
landtype (non-oceanic)	0.3561	0.0930	3.8280	0.00013
landtype (island null)	-2.1220	0.0564	-37.6550	<2.00E-16
landtype (oceanic)	-1.7330	0.0701	-24.7350	<2.00E-16
absolute latitude	-0.0206	0.0012	-17.5320	<2.00E-16
spatial autocorrelation	0.9504	0.0142	67.0000	<2.00E-16
landtype (non-oceanic)*absolute latitude	-0.0241	0.0023	-10.5620	<2.00E-16
landtype (island null)*absolute latitude	0.0000	0.0017	-0.0040	0.99702
landtype (oceanic)*absolute latitude	0.0069	0.0024	2.8470	0.00441

SI Table 2 | Model results for drivers of island species deficit

Results for models used to test for drivers of island species deficit, including the influence of abiotic variables and the mutualism filter strength, including model averaging (A) and model selection via testing increasing interactions (B,C). The variable 'mutualism filter strength' represents the proportion of mainland floras associating with any one of the three mutualisms included in our analyses (biotic pollination, arbuscular mycorrhizal, or N-fixing). Statistical analysis used Satterthwaite's approximations for t-test and corresponding p values.

A. Model averaging				
	Estimate	Std. Error	z value	p value
(Intercept)	1225.5083	115.7658	10.5240	<2.00E-16

absolute latitude	-369.6273	37.8687	9.7030	<2.00E-16
area	-456.2335	34.9521	12.9780	<2.00E-16
distance	596.3570	87.4566	6.7790	<2.00E-16
precipitation	-99.7549	19.5658	5.0680	0.00000
elevation range	-13.1875	23.2206	0.5650	0.57200
mutualism filter strength	208.5520	37.3913	5.5450	<2.00E-16
spatial autocorrelation	1.0001	0.0659	15.0950	<2.00E-16
B. Model selection (df = 199)				
	Estimate	Std. Error	t value	p value
(Intercept)	1068.3288	115.2197	9.2720	<2.00E-16
mutualism filter strength	123.7825	133.0032	0.9310	0.35315
area	-475.8678	38.0561	-12.5040	<2.00E-16
distance	569.6707	85.8393	6.6360	0.00000
precipitation	-92.5052	20.6672	-4.4760	0.00001
elevation range	2.2104	22.5097	0.0980	0.92187
absolute latitude	-382.4319	70.3613	-5.4350	0.00000
rac	1.0125	0.0695	14.5620	<2.00E-16
mutualism filter strength*area	-205.6436	60.2520	-3.4130	0.00078
mutualism filter strength*dist	210.7617	82.8699	2.5430	0.01174
mutualism filter strength*absolute latitude	-175.7389	29.7839	-5.9000	0.00000
area*absolute latitude	-84.2439	61.6585	-1.3660	0.17339
preciptation*absolute latitude	46.4627	24.5663	1.8910	0.06003

C. Model selection

Model (final model in bold)	AIC
mutualism filter strength*area + distance + precitation + elevation range + absolute latitude	2980.4910
mutualism filter strength*area + mutualism filter strength*distance + precitation + elevation range + absolute latitude	2976.1720
mutualism filter strength*area + mutualism filter strength*distance + mutualism filter strength*prec + elevation range + absolute latitude	2981.3770

mutualism filter strength*area + mutualism filter strength*distance + precitation + mutualism filter strength*elevation range + absolute latitude	2979.6830
mutualism filter strength*area + mutualism filter strength*distance +precitation + elevation range + mutualism filter strength*absolute latitude	2974.2880
mutualism filter strength*area + mutualism filter strength*distance + precitation + elevation range + mutualism filter strength*absolute latitude + abslatitude*area	2973.9770
mutualism filter strength*area + mutualism filter strength*distance + precitation + elevation range + mutualism filter strength*absolute latitude + absolute latitude*area + absolute latitude*distance	2980.5340
**mutualism filter strength*area + mutualism filter strength*distance + precitation + elevation range + mutualism filter strength*absolute latitude + absolute latitude*area + absolute latitude*prec**	**2971.7110**
biotic.ml*area + biotic.ml*distance + precitation + elevation range + biotic.ml*abslatitude + absolute latitude*area + absolute latitude*precitation + absolute latitude*elevation range	2973.5310

SI Table 3 | Model results for proportional specific deficit and contribution to species deficit for pollination mutualisms

Model results for GLMs testing drivers of proportional species deficit and contribution to species deficit within the pollination mutualism as well as contribution to species deficit within mutualist status to include interactions with distance, area, and precipitation (removed in final model if non-significant). Models are run for dataset in which extremes were constrained to 0 or 1 ("constrained response model", reported in main models, A.), or where extremes were kept ("extreme response model", B.). Statistical analysis used Satterthwaite's approximations for t-test and corresponding p values.

**A. Constrained response model**				
Proportional species deficit (df = 415)				
Variable	Estimate	Std. Error	t value	p value
(Intercept)	0.2735	0.0492	5.5590	0.00000

absolute latitude	0.0016	0.0011	1.3890	0.16600
pollination syndrome (biotic)	0.1861	0.0410	4.5440	0.00001
area	-0.1598	0.0094	-16.9770	<2.00E-16
distance	0.2043	0.0205	9.9490	<2.00E-16
elevation range	0.0078	0.0054	1.4580	0.14600
precipitation	-0.0375	0.0044	-8.4830	0.00000
spatial autocorrelation	0.0433	0.0022	19.4370	<2.00E-16
absolute latitude*pollination syndrome (biotic)	-0.0012	0.0012	-0.9950	0.32000
Contribution to species deficit (df = 413)				
(Intercept)	0.0277	0.0034	8.2350	0.00000
absolute latitude(1)	-0.0023	0.0002	-14.8330	<2.00E-16
absolute latitude(2)	0.0001	0.0000	41.9310	<2.00E-16
pollination syndrome (biotic)	0.9446	0.0024	401.2740	<2.00E-16
area	0.0000	0.0009	0.0000	1.00000
distance	0.0000	0.0021	0.0000	1.00000
elevation range	0.0000	0.0005	0.0000	1.00000
precipitation	0.0000	0.0004	0.0000	1.00000
spatial autocorrelation	-0.0382	0.0009	-42.6660	<2.00E-16
absolute latitude(1)*pollination syndrome (biotic)	0.0046	0.0002	21.1570	<2.00E-16
absolute latitude(2)*pollination syndrome (biotic)	-0.0002	0.0000	-59.4430	<2.00E-16
Biotic contribution to species deficit (df = 208)				
(Intercept)	0.9709	0.0035	278.6500	<2.00E-16
absolute latitude(1)	0.0022	0.0003	6.8890	0.00000
absolute latitude(2)	-0.0001	0.0000	-19.6590	<2.00E-16
spatial autocorrelation	0.0238	0.0035	6.9000	0.00000
Abiotic contribution to species deficit (208)				
(Intercept)	0.0291	0.0035	8.3600	0.00000
absolute latitude(1)	-0.0022	0.0003	-6.8890	0.00000
absolute latitude(2)	0.0001	0.0000	19.6590	<2.00E-16
spatial autocorrelation	0.0238	0.0035	6.9000	0.00000
**B. Extreme response model**				
Proportional species deficit (df = 381)				
Variable	Estimate	Std. Error	t value	p value
(Intercept)	0.4997	0.0344	14.5430	<2.00E-16
absolute latitude	0.0020	0.0008	2.5260	0.01200
pollination syndrome (biotic)	0.1798	0.0280	6.4330	0.00000

area	-0.0642	0.0071	-9.0910	<2.00E-16
distance	0.1332	0.0140	9.5160	<2.00E-16
elevation range	-0.0033	0.0036	-0.9100	0.36300
precipitation	-0.0239	0.0031	-7.6800	0.00000
spatial autocorrelation	0.0351	0.0020	17.3640	<2.00E-16
absolute latitude*pollination syndrome (biotic)	-0.0019	0.0008	-2.3360	0.02000
Contribution to species deficit (df = 379)				
(Intercept)	0.0253	0.0017	15.2300	<2.00E-16
absolute latitude(1)	-0.0019	0.0001	-25.9500	<2.00E-16
absolute latitude(2)	0.0001	0.0000	83.9600	<2.00E-16
pollination syndrome (biotic)	0.9493	0.0011	861.9900	<2.00E-16
area	0.0000	0.0005	0.0000	1.00000
distance	0.0000	0.0010	0.0000	1.00000
elevation range	0.0000	0.0003	0.0000	1.00000
precipitation	0.0000	0.0002	0.0000	1.00000
spatial autocorrelation	-0.0374	0.0005	-72.1200	<2.00E-16
absolute latitude(1)*pollination syndrome (biotic)	0.0038	0.0001	37.0600	<2.00E-16
absolute latitude(2)*pollination syndrome (biotic)	-0.0002	0.0000	-119.4100	<2.00E-16
Biotic contribution to species deficit (df = 191)				
(Intercept)	0.9752	0.0022	442.6010	<2.00E-16
absolute latitude(1)	0.0018	0.0002	8.8790	0.00000
absolute latitude(2)	-0.0001	0.0000	-29.3870	<2.00E-16
spatial autocorrelation	0.0349	0.0028	12.5650	<2.00E-16
Abiotic contribution to species deficit (df = 191)				
(Intercept)	0.0248	0.0022	11.2350	<2.00E-16
absolute latitude(1)	-0.0018	0.0002	-8.8790	0.00000
absolute latitude(2)	0.0001	0.0000	29.3870	<2.00E-16
spatial autocorrelation	0.0349	0.0028	12.5650	<2.00E-16

SI Table 4 | Model results for proportional specific deficit and contribution to species deficit for mycorrhizal mutualisms.

Model results for GLMs testing drivers of proportional species deficit and contribution to species deficit within the mycorrhizal mutualism as well as contribution to species deficit within

mutualist status to include interactions with distance, area, and precipitation (removed in final model if non-significant). Models are run for dataset in which extremes were constrained to 0 or 1 ("constrained response model", reported in main models, A.), or where extremes were kept ("extreme response model", B.). Statistical analysis used Satterthwaite's approximations for t-test and corresponding p values.

**A. Constrained response model**				
Proportional species deficit (df = 835)				
Variable	Estimate	Std. Error	t value	p value
(Intercept)	0.4107	0.0240	17.1390	<2e-16
absolute latitude	0.0002	0.0006	0.3710	0.71100
mycorrhizal type (AM)	0.0242	0.0193	1.2550	0.20990
mycorrhizal type (EM)	0.0944	0.0392	2.4110	0.01610
mycorrhizal type (ORC)	0.0301	0.0253	1.1880	0.23530
area	-0.1762	0.0049	-35.6120	<2e-16
distance	0.2030	0.0108	18.7770	<2e-16
elevation range	-0.0073	0.0028	-2.5750	0.01020
precipitation	-0.0471	0.0023	-20.2630	<2e-16
spatial autocorrelation	0.0571	0.0016	35.8950	<2e-16
absolute latitude*mycorrhizal type (AM)	-0.0001	0.0007	-0.2010	0.84110
absolute latitude*mycorrhizal type (EM)	-0.0012	0.0014	-0.9050	0.36570
absolute latitude*mycorrhizal type (ORC)	0.0001	0.0012	0.0640	0.94900
Contribution to species deficit (df = 831)				
(Intercept)	0.0384	0.0034	11.2830	<2.00E-16
absolute latitude(1)	-0.0007	0.0002	-3.6520	0.00028
absolute latitude(2)	0.0001	0.0000	16.3800	<2.00E-16
myctypeAM	0.8123	0.0030	269.0540	<2.00E-16
myctypeEM	-0.0270	0.0030	-8.9440	<2.00E-16
myctypeORC	0.0648	0.0030	21.4650	<2.00E-16
area	0.0003	0.0009	0.4020	0.68797
distance	-0.0002	0.0019	-0.0810	0.93507
elevation range	0.0001	0.0005	0.1110	0.91167
precipitation	0.0001	0.0004	0.2000	0.84175
spatial autocorrelation	-0.0004	0.0002	-2.4870	0.01307
absolute latitude(1)*mycorrhizal type (AM)	0.0047	0.0003	16.5840	<2.00E-16
absolute latitude(2)*mycorrhizal type (AM)	-0.0002	0.0000	-30.7200	<2.00E-16

absolute latitude(1)*mycorrhizal type (EM)	0.0009	0.0003	3.1670	0.00160
absolute latitude(2)*mycorrhizal type (EM)	-0.0001	0.0000	-10.1310	<2.00E-16
absolute latitude(1)*mycorrhizal type (ORC)	-0.0027	0.0003	-9.6190	<2.00E-16
absolute latitude(2)*mycorrhizal type (ORC)	0.0000	0.0000	-5.2810	0.00000
AM contribution to species deficit (df = 206)				
(Intercept)	0.8485	0.0027	319.7020	<2.00E-16
absolute latitude(1)	0.0033	0.0003	10.8260	<2.00E-16
absolute latitude(2)	-0.0001	0.0000	-13.0590	<2.00E-16
spatial autocorrelation	0.0009	0.0009	1.0220	0.30800
absolute latitude(1)*area	-0.0008	0.0002	-4.4770	0.00001
absolute latitude(2)*area	0.0000	0.0000	4.9150	0.00000
EM contribution to species deficit (df = 206)				
(Intercept)	0.0174	0.0005	34.8040	<2.00E-16
spatial autocorrelation	-0.0004	0.0002	-2.0520	0.04140
absolute latitude(1)*precipitation	-0.0003	0.0001	-5.5700	0.00000
absolute latitude(2)*precipitation	0.0000	0.0000	6.9270	0.00000
ORC contribution to species deficit (df = 208)				
(Intercept)	0.0766	0.0023	33.8610	<2.00E-16
spatial autocorrelation	-0.0028	0.0006	-4.9310	0.00000
absolute latitude(1)*area	0.0010	0.0002	5.6100	0.00000
absolute latitude(2)*area	0.0000	0.0000	0.1250	0.90100
NM contribution to species deficit (df = 208)				
(Intercept)	0.0396	0.0021	19.2040	<2.00E-16
spatial autocorrelation	0.0010	0.0003	3.2420	0.00138
absolute latitude(1)*distance	-0.0006	0.0001	-4.4300	0.00002
absolute latitude(2)*distance	0.0000	0.0000	17.5510	<2.00E-16
**B. Extreme response model**				
Proportional species deficit (df = 799)				
Variable	Estimate	Std. Error	t value	p value
(Intercept)	0.4781	0.0205	23.3260	<2.00E-16
absolute latitude	0.0003	0.0005	0.4590	0.64601
mycorrhizal type (AM)	0.0214	0.0164	1.3030	0.19294
mycorrhizal type (EM)	0.0987	0.0333	2.9640	0.00313
mycorrhizal type (NM)	0.0482	0.0214	2.2500	0.02474
area	-0.1169	0.0046	-25.1540	<2.00E-16
distance	0.2017	0.0091	22.2590	<2.00E-16
elevation range	-0.0154	0.0024	-6.4310	0.00000
precipitation	-0.0403	0.0020	-20.4550	<2.00E-16

spatial autocorrelation	0.0581	0.0019	31.1940	<2.00E-16
absolute latitude*mycorrhizal type (AM)	0.0000	0.0006	-0.0200	0.98428
absolute latitude*mycorrhizal type (EM)	-0.0013	0.0012	-1.0910	0.27577
absolute latitude*mycorrhizal type (ORC)	-0.0001	0.0010	-0.0720	0.94257
Contribution to species deficit (df = 795)				
(Intercept)	0.0385	0.0015	26.3260	<2.00E-16
absolute latitude(1)	-0.0006	0.0001	-7.0640	0.00000
absolute latitude(2)	0.0001	0.0000	35.0900	<2.00E-16
myctypeAM	0.8143	0.0013	605.8070	<2.00E-16
myctypeEM	-0.0277	0.0013	-20.6440	<2.00E-16
myctypeORC	0.0640	0.0013	47.6720	<2.00E-16
area	0.0000	0.0005	0.0730	0.94100
distance	-0.0006	0.0008	-0.6660	0.50600
elevation range	-0.0001	0.0002	-0.3640	0.71600
precipitation	0.0003	0.0002	1.4540	0.14600
spatial autocorrelation	-0.0445	0.0016	-28.6300	<2.00E-16
absolute latitude(1)*mycorrhizal type (AM)	0.0045	0.0001	35.8340	<2.00E-16
absolute latitude(2)*mycorrhizal type (AM)	-0.0002	0.0000	-67.4010	<2.00E-16
absolute latitude(1)*mycorrhizal type (EM)	0.0009	0.0001	7.2470	0.00000
absolute latitude(2)*mycorrhizal type (EM)	-0.0001	0.0000	-22.3480	<2.00E-16
absolute latitude(1)*mycorrhizal type (ORC)	-0.0028	0.0001	-22.3450	<2.00E-16
absolute latitude(2)*mycorrhizal type (ORC)	0.0000	0.0000	-10.4390	<2.00E-16
AM contribution to species deficit (df = 197)				
(Intercept)	0.8502	0.0014	591.4350	<2.00E-16
absolute latitude(1)	0.0040	0.0002	21.9860	<2.00E-16
absolute latitude(2)	-0.0001	0.0000	-26.1170	<2.00E-16
spatial autocorrelation	0.0176	0.0028	6.3110	0.00000
absolute latitude(1)*area	0.0000	0.0001	0.0070	0.99400
absolute latitude(2)*area	0.0000	0.0000	0.0940	0.92500
EM contribution to species deficit (df = 199)				
(Intercept)	0.0174	0.0005	36.7050	<2.00E-16
spatial autocorrelation	0.0270	0.0051	5.3080	0.00000
absolute latitude(1)*precipitation	-0.0003	0.0001	-5.9530	0.00000
absolute latitude(2)*precipitation	0.0000	0.0000	7.5120	0.00000
ORC contribution to species deficit (df = 199)				
(Intercept)	0.0748	0.0023	33.0920	<2.00E-16
spatial autocorrelation	0.0041	0.0076	0.5420	0.58800
absolute latitude(1)*area	0.0008	0.0002	4.4800	0.00001

absolute latitude(2)*area	0.0000	0.0000	1.3260	0.18600
NM contribution to species deficit (df = 199)				
(Intercept)	0.0407	0.0017	23.6590	<2.00E-16
spatial autocorrelation	0.0243	0.0030	8.0340	0.00000
absolute latitude(1)*distance	-0.0007	0.0001	-6.0290	0.00000
absolute latitude(2)*distance	0.0001	0.0000	21.5720	<2.00E-16

SI Table 5 | Model results for proportional specific deficit and contribution to species deficit for N-fixing mutualism

Model results for GLMs testing drivers of proportional species deficit and contribution to species deficit within the N-fixing mutualism as well as contribution to species deficit within mutualist status to include interactions with distance, area, and precipitation (removed in final model if non-significant). Models are run for dataset in which extremes were constrained to 0 or 1 ("constrained response model", reported in main models, A.), or where extremes were kept ("extreme response model", B.). Statistical analysis used Satterthwaite's approximations for t-test and corresponding p values.

**A. Constrained response model**				
Proportional species deficit (df = 415)				
Variable	Estimate	Std. Error	t value	p value
(Intercept)	0.4158	0.0275	15.1230	<2.00E-16
absolute latitude	0.0005	0.0004	1.2560	0.20970
N-fixing type (N-fixing)	0.0343	0.0150	2.2850	0.02280
area	-0.1399	0.0084	-16.7460	<2.00E-16
distance	0.2188	0.0184	11.9220	<2.00E-16
elevation range	-0.0049	0.0048	-1.0090	0.31350
precipitation	-0.0268	0.0041	-6.5920	0.00000
spatial autocorrelation	0.0564	0.0024	23.1200	<2.00E-16
absolute latitude*N-fixing type (N-fixing)	0.0013	0.0006	2.0610	0.03990
Contribution to species deficit (df = 411)				
(Intercept)	0.5318	0.0050	106.6300	<2.00E-16
absolute latitude (1)	-0.0196	0.0004	-45.7500	<2.00E-16
absolute latitude (2)	0.0008	0.0000	43.4100	<2.00E-16

absolute latitude (3)	0.0000	0.0000	-27.4100	<2.00E-16
N-fixing type (N-fixing)	-0.0636	0.0042	-15.0900	<2.00E-16
area	0.0000	0.0014	0.0000	1.00000
distance	0.0000	0.0029	0.0000	1.00000
elevation range	0.0000	0.0008	0.0000	1.00000
precipitation	0.0000	0.0006	0.0000	1.00000
spatial autocorrelation	-0.0525	0.0008	-64.7400	<2.00E-16
absolute latitude (1)*N-fixing type (N-fixing)	0.0392	0.0006	64.8300	<2.00E-16
absolute latitude (2)*N-fixing type (N-fixing)	-0.0016	0.0000	-61.9000	<2.00E-16
absolute latitude (3)*N-fixing type (N-fixing)	0.0000	0.0000	39.1900	<2.00E-16
N-fixing contribution to species deficit (df = 207)				
(Intercept)	0.4616	0.0087	53.0590	<2.00E-16
absolute latitude (1)	0.0157	0.0016	9.7990	<2.00E-16
absolute latitude (2)	-0.0006	0.0001	-6.9810	0.00000
absolute latitude (3)	0.0000	0.0000	3.3650	0.00091
spatial autocorrelation	0.0584	0.0068	8.5780	0.00000
absolute latitude(1)*area	-0.0041	0.0012	-3.4110	0.00078
absolute latitude(2)*area	0.0002	0.0001	2.9060	0.00406
absolute latitude(3)*area	0.0000	0.0000	-2.5370	0.01193
Non N-fixing contribution to species deficit (df = 204)				
(Intercept)	0.5384	0.0087	61.8960	<2.00E-16
absolute latitude (1)	-0.0157	0.0016	-9.7990	<2.00E-16
absolute latitude (2)	0.0006	0.0001	6.9810	0.00000
absolute latitude (3)	0.0000	0.0000	-3.3650	0.00091
spatial autocorrelation	0.0584	0.0068	8.5780	0.00000
absolute latitude(1)*area	0.0041	0.0012	3.4110	0.00078
absolute latitude(2)*area	-0.0002	0.0001	-2.9060	0.00406
absolute latitude(3)*area	0.0000	0.0000	2.5370	0.01193
**B. Extreme response model**				
Proportional species deficit (df = 411)				
Variable	Estimate	Std. Error	t value	p value
(Intercept)	0.4486	0.0263	17.0610	<2.00E-16
absolute latitude	0.0007	0.0004	1.9080	0.05710
N-fixing type (N-fixing)	0.0371	0.0143	2.6020	0.00960
area	-0.1044	0.0083	-12.5900	<2.00E-16
distance	0.2177	0.0174	12.5360	<2.00E-16
elevation range	-0.0118	0.0046	-2.5700	0.01050
precipitation	-0.0244	0.0039	-6.3310	0.00000
spatial autocorrelation	0.0555	0.0025	22.4400	<2.00E-16

absolute latitude*N-fixing type (N-fixing)	0.0011	0.0006	1.9020	0.05790
Contribution to species deficit (df = 407)				
(Intercept)	0.5311	0.0036	146.0500	<2.00E-16
absolute latitude (1)	-0.0201	0.0003	-65.3000	<2.00E-16
absolute latitude (2)	0.0008	0.0000	62.3800	<2.00E-16
absolute latitude (3)	0.0000	0.0000	-40.1600	<2.00E-16
N-fixing type (N-fixing)	-0.0622	0.0030	-20.5400	<2.00E-16
area	0.0000	0.0010	0.0000	1.00000
distance	0.0000	0.0021	0.0000	1.00000
elevation range	0.0000	0.0005	0.0000	1.00000
precipitation	0.0000	0.0005	0.0000	1.00000
spatial autocorrelation	-0.0641	0.0008	-76.5800	<2.00E-16
absolute latitude (1)*N-fixing type (N-fixing)	0.0402	0.0004	92.4900	<2.00E-16
absolute latitude (2)*N-fixing type (N-fixing)	-0.0017	0.0000	-88.8900	<2.00E-16
absolute latitude (3)*N-fixing type (N-fixing)	0.0000	0.0000	57.3600	<2.00E-16
N-fixing contribution to species deficit (df = 205)				
(Intercept)	0.4730	0.0056	84.6520	<2.00E-16
absolute latitude (1)	0.0220	0.0011	20.3640	<2.00E-16
absolute latitude (2)	-0.0009	0.0001	-16.0430	<2.00E-16
absolute latitude (3)	0.0000	0.0000	10.0800	<2.00E-16
spatial autocorrelation	0.0688	0.0045	15.1800	<2.00E-16
absolute latitude(1)*area	0.0026	0.0008	3.2060	0.00156
absolute latitude(2)*area	-0.0001	0.0001	-2.4180	0.01649
absolute latitude(3)*area	0.0000	0.0000	2.0210	0.04463
Non N-fixing contribution to species deficit (df = 205)				
(Intercept)	0.5270	0.0056	94.3140	<2.00E-16
absolute latitude (1)	-0.0220	0.0011	-20.3640	<2.00E-16
absolute latitude (2)	0.0009	0.0001	16.0430	<2.00E-16
absolute latitude (3)	0.0000	0.0000	-10.0800	<2.00E-16
spatial autocorrelation	0.0688	0.0045	15.1800	<2.00E-16
absolute latitude(1)*area	-0.0026	0.0008	-3.2060	0.00156
absolute latitude(2)*area	0.0001	0.0001	2.4180	0.01649
absolute latitude(3)*area	0.0000	0.0000	-2.0210	0.04463
